# Polio Outbreak Among Nomads in Chad: Outbreak Response and Lessons Learned

**DOI:** 10.1093/infdis/jit564

**Published:** 2013-10-23

**Authors:** Serigne M. Ndiaye, Mahamat Abdoulaye Ahmed, Melinda Denson, Allen S. Craig, Katrina Kretsinger, Baharadine Cherif, Pierre Kandolo, Daugla Doumagoum Moto, Ayangma Richelot, Jude Tuma

**Affiliations:** 1 Global Immunization Division, Centers for Disease Control and Prevention, Atlanta, Georgia;; 2 Centre de Support en Santé International; 3 Programme Elargie ide Vaccination, Ministere de la Santé Publique;; 4 World Health Organization, N’Djamena, Chad

**Keywords:** nomad health, vaccinations of mobile populations, vaccination outreach strategies, polio vaccination

## Abstract

**Background.:**

In response to the 2011 and 2012 polio epidemic in Chad, Chad’s Ministry of Public Health, with support from Global Polio Eradication Initiative partners, took steps to increase vaccination coverage of nomadic children with targeted polio campaigns. This article describes the strategies we used to vaccinate nomads in 3 districts of Chad.

**Methods.:**

Our targeted interventions involved using mobile vaccination teams, recruiting local nomads to identify settlements, using social mobilization, and offering vaccinations to children, women, and animals.

**Results.:**

Vaccination coverage of nomadic children 0–59 months of age increased, particularly among those never before vaccinated against polio. These increases occurred mostly in the intervention districts of Dourbali, from 2956 to 8164 vaccinated children, and Kyabe, from 7319 to 15 868. The number of first-time vaccinated nomadic children also increased the most in these districts, from 60 to 131 in Dourbali and from 1302 to 2973 in Kyabe. Coverage in the Massaguet district was only 37.7%.

**Conclusions.:**

Our success was probably due to (1) appointment of staff to oversee implementation, (2) engagement of the national government and its partners, (3) participation of nomadic community leaders, (4) intersectoral collaboration between human and animal health services, and (5) flexibility and capacity of vaccinators to vaccinate when and where nomads were available.

In the African region, Angola, the Democratic Republic of Congo, and Chad succeeded in stopping circulation of wild poliovirus (WPV) type 1 (WPV1) and type 3 (WPV3) in 2012. Chad was the last among them with onset of the last case on 14 June 2012. Chad faces many challenges in its goal to eventually eradicate polio, including its poor health infrastructure and the risk for importation of polioviruses from Nigeria, the only polio-endemic country in Africa, where WPV strains still circulate. A significant challenge has been the difficulty in improving immunization coverage of the relatively small nomadic populations, whose rate of polio infection is disproportionately high, accounting for 12% and 40% of cases in 2011 and 2012, even though they represent between 3.5% and 6% of the population. In 2012, Chad made significant progress in reaching these high-risk populations. For example, nomadic children were targeted for the first time during supplemental immunization activities (SIAs) in selected regions in June 2012. This article details the strategy we used to improve polio vaccination coverage among nomads in the districts of Kyabe, Dourbali, and Massaguet during polio vaccination campaigns.

## EPIDEMIOLOGIC CONTEXT

Polio transmission was stopped in Chad in 2000 but was reestablished in 2007 after importation of the WPV3 from Nigeria and a rise in the number of polio cases to 64. In 2010, WPV1 transmission was reestablished after importation from Nigeria [1]. The polio outbreak reached its peak in 2011 with 132 confirmed cases (129 WPV1 and 3 WPV3). The 2011 epidemic was concentrated mainly in the southern regions of Logone Occidental and Logone Oriental. Children 12–59 months of age were most affected, with 79% of the polio cases, followed by children 5–14 years and infants 0–11 months of age. In 2011 and 2012, respectively, 57% and 80% of polio cases occurred in boys. Polio cases declined by 96% in 2012 [2].

Despite a low routine third dose of oral polio vaccine coverage estimated at about 31% in 2011, Chad has improved polio population immunity and has stopped transmission of WPV since June 2012 by implementing more than a dozen national immunization days and multiple subnational immunization days since January 2011. Nonpolio acute flaccid paralysis vaccination data show that in the fourth quarter of 2011, 55% of children received ≥4 doses of oral polio vaccine; in 2012, this proportion increased to 59%. [3].

## NOMADIC HEALTH CARE AND RISKS FOR POLIO TRANSMISSION IN CHAD

Nomadic populations are vulnerable to diseases because of their limited access to healthcare services compared with the general population. Mistrust of health services [4] and preferences for traditional medicine make it less likely that nomads will access health services [5]. Other factors include sociolinguistic barriers [6] and discrimination [7]. Integrating nomads into systems of disease surveillance has also proved difficult [8], as has reaching them with immunization services [9].

Because nomads in general live in rural and isolated settlements, they have fewer opportunities to access healthcare services, which are typically concentrated in urban settlements. The constant mobility of nomads restricts access, particularly where extended courses of treatment, such as delivery of childhood immunizations, is indicated [10]. A substantial body of evidence shows effective interventions to reach nomadic populations with health services (Table 1). The isolated lifestyle of nomads that limits their access to health services also may have contributed to the transmission of communicable diseases such as measles, and perhaps polio remaining low among them [11, 12], especially before they began to settle in populated areas in the 1990s and 2000s as a result of long years of drought in the African Sahel region. With changes in rainfall in the African Sahel countries, and the ensuing drought, nomads have progressively been settling in more populated areas where they can find food and water for themselves and their animals. As a result, their increased contact with the general population has provided fertile ground for epidemics.

**Figure 1.**
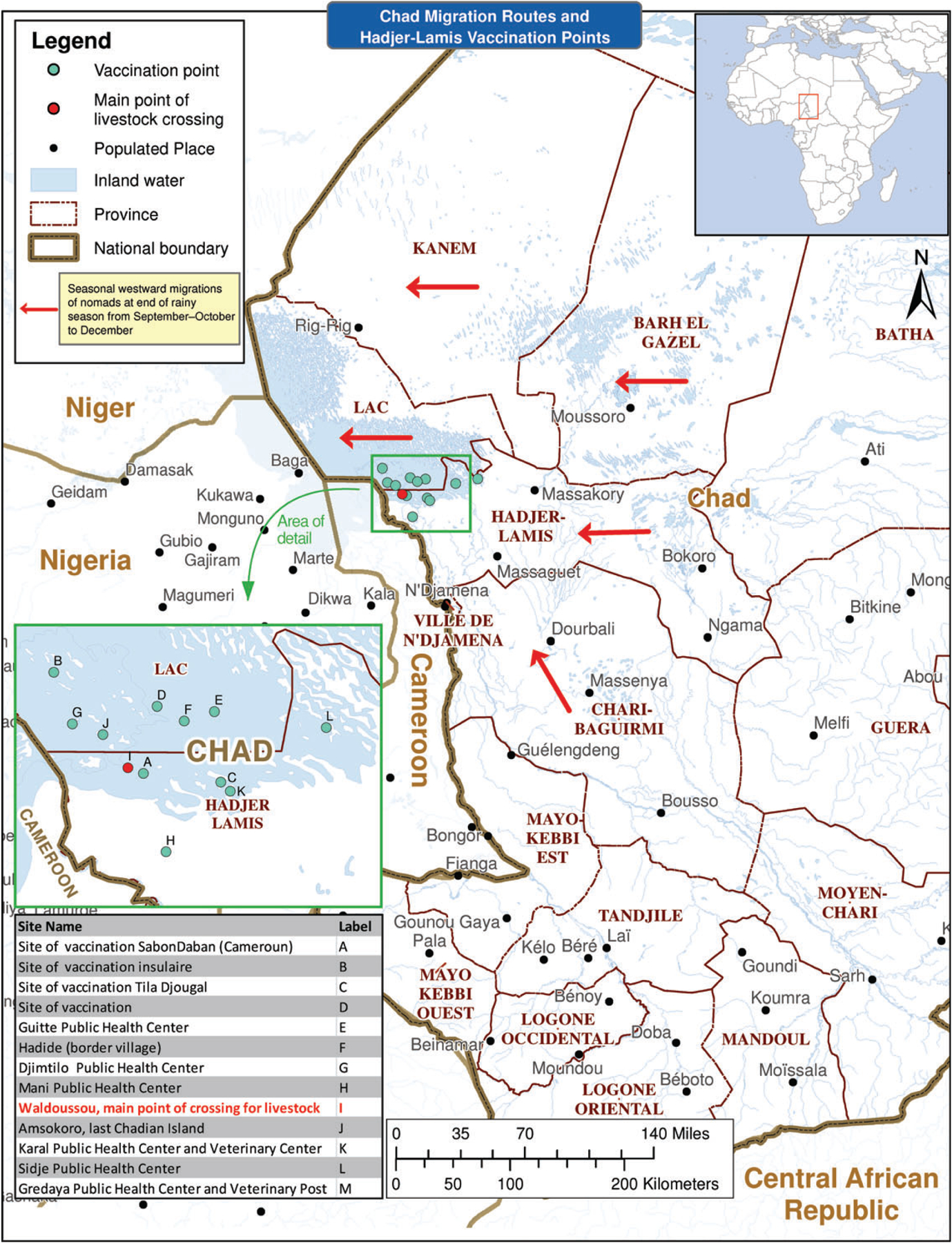
Chad migration routes and Hadjer-Lamis vaccination points.

The relative proportion of the nomadic population as a percentage of the total population in Chad has decreased from 6% in 1992 to about 3.5% in 2009; however, the absolute numbers have grown from an estimate of 83 500 in 1992 [13] to about 361 552, according to the 2009 country population census [14]. In western Chad, nomads migrate seasonally in a predictable northerly and easterly direction, generally from April–May before onset of the rainy season in June; they follow the opposite direction beginning at the end of the rainy season around September–October through December (Figure 1). During migratory periods, nomads follow corridors where they have informants along their routes, as well as permanent posts where they can stop occasionally to resupply basic food and take care of other business as needed.

To our knowledge, no current national data are available on vaccination coverage among nomadic children in Chad. Estimates from small and localized studies suggest vaccination coverage is generally low. According to Daoud et al, 2000, there were no fully immunized nomadic children in the Chari-Baguirimi and Kanem regions in 2000 [15]. Schelling et al [16] estimated coverage of the third doses of diphtheria-tetanus-pertussis and polio vaccines among nomadic infants 0–11 months of age to be 8% in 2003 and 14% in 2004 in the subdistrict of Gredaya and 8% and 7% in 2003 and 2004 in the community of Dourbali.

**Figure 2.**
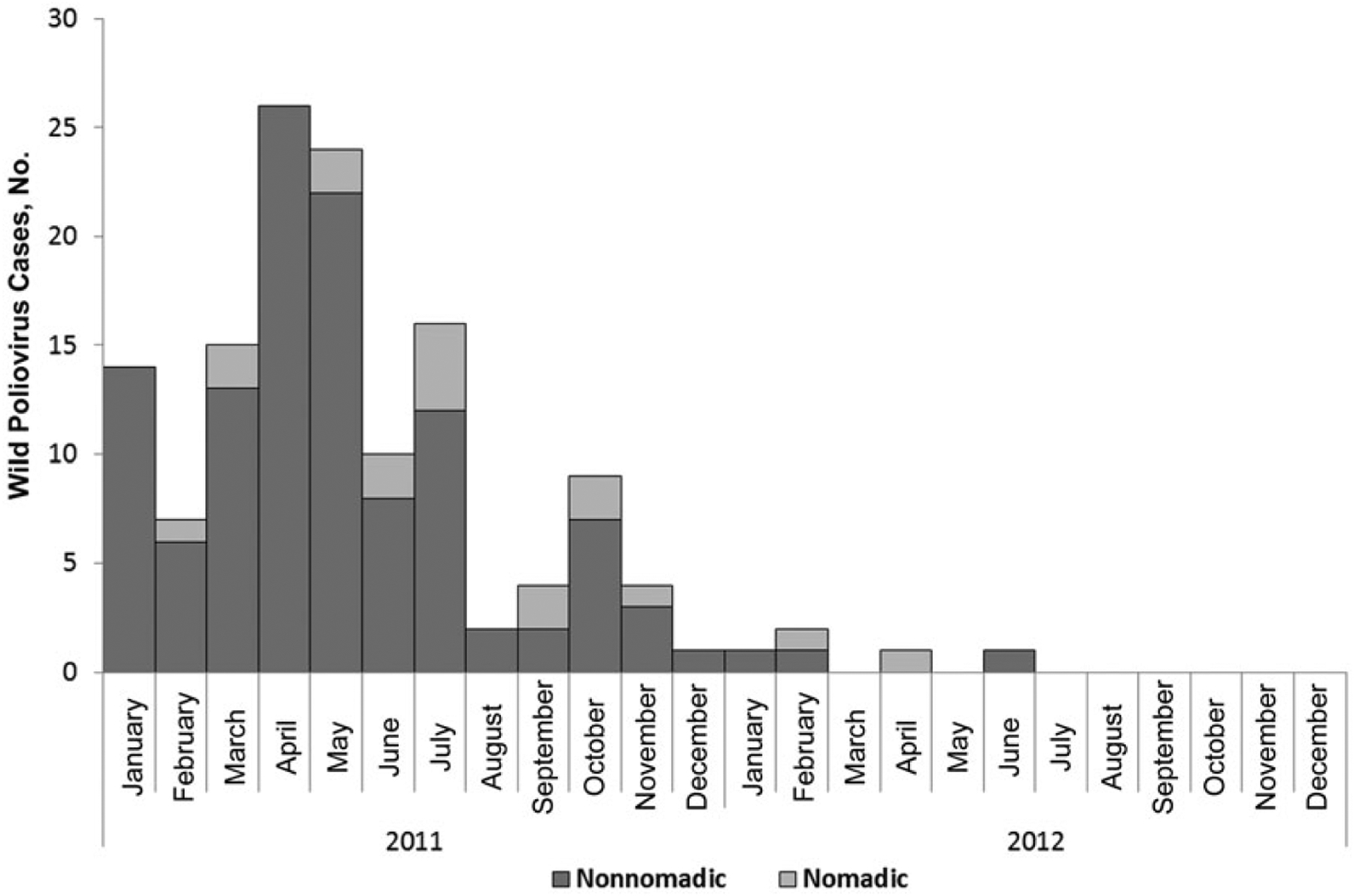
Wild poliovirus cases among nomads and the general population in Chad, 2011–2012.

**Figure 3.**
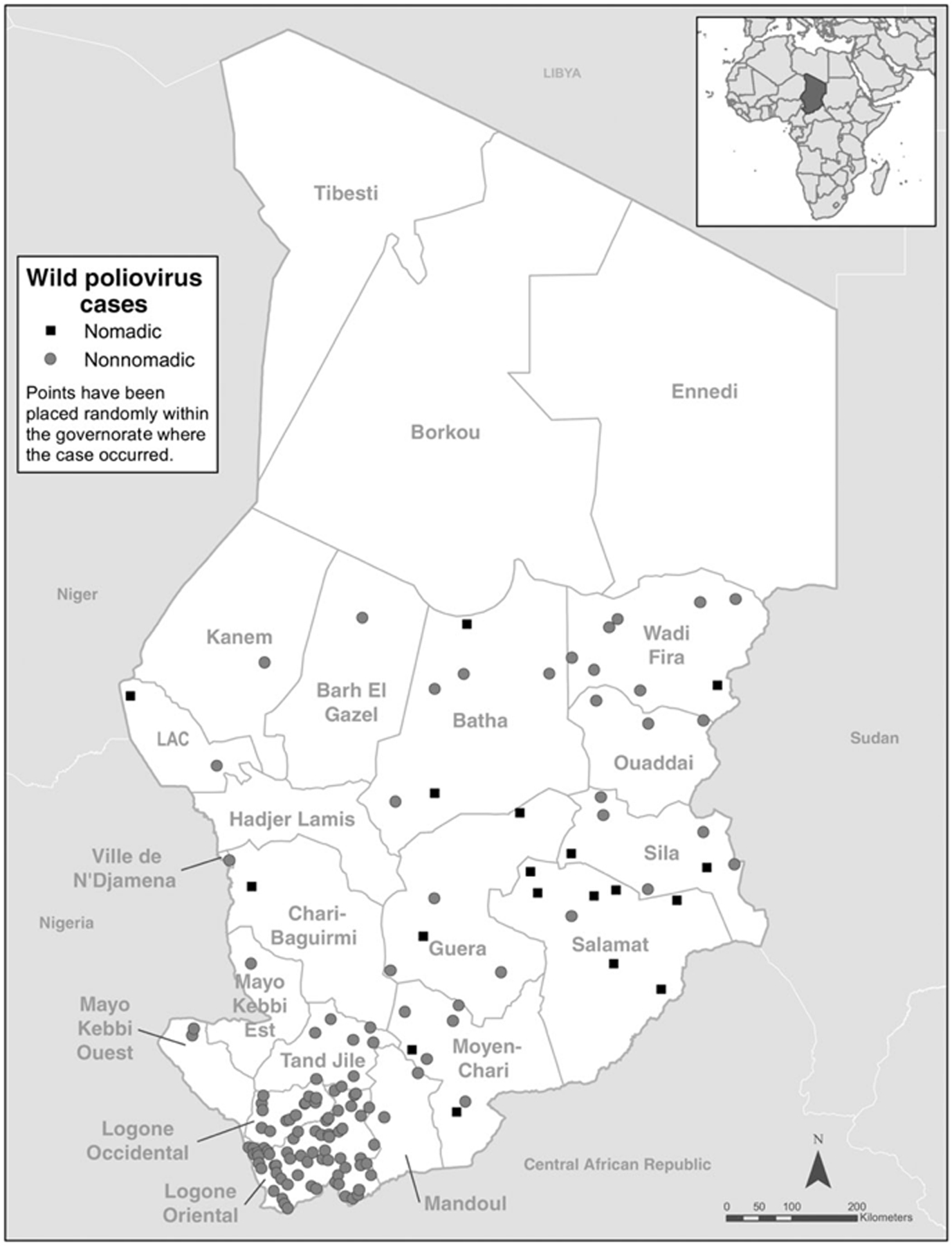
Distribution of wild poliovirus cases among regions in Chad, 2011–2012.

The polio epidemic in Chad has significantly affected the nomadic populations in large proportions relative to their size. Figure 2 shows the number of WPV cases reported by month and throughout 2011 and 2012. In total, 132 cases of polio were reported in 2011, including 116 among the general population. Five polio cases were reported in 2012, including 3 among the general population. Overall, 12% of confirmed WPV cases in 2011 (16 of 132) and 40% of cases as of May 2012 (2 of 5) occurred among nomadic children, with peak periods in March, May–July, and September–November 2011. Similarly to the general population, most polio cases among nomads (11 of 16; 69%) occurred in boys. Among nomads, older children (5–14 years of age) are most affected (50% of polio cases), whereas in Chad’s general population children 12–59 months of age are most affected (about 79% of cases). The geographic distribution of WPV cases across Chad is shown in Figure 3.

## METHODS

### Establishing National and Stakeholder Partnership and Support

After the peak of the polio epidemic in 2011, a renewed national awareness and determination to eradicate polio in Chad emerged. Chad’s president, Idriss Deby Itno, issued orders to regional governors asking them to engage in and be supportive of polio eradication activities. This national commitment increased the pressure on the Ministry of Health’s Expanded Program on Immunization (EPI) to improve vaccinations of nomads, whose polio epidemic burden was alarming. As data became available, Global Polio Eradication Initiative in-country partners also perceived the need to address the issues and pledged to provide increased support.

To assist with this effort, a team of scientists at the US Centers for Disease Control and Prevention conducted a systematic review of interventions targeting nomads with health services and proposed a menu of intervention options to the EPI in Chad. An important review finding was the importance of integrating other services with immunization and establishing intersectoral collaboration, especially with veterinary services, to more effectively gain interest and participation among nomads. As a result, we decided to fully collaborate with the Centre de Support en Santé International, a national center specializing in rural health with many years of experience in projects directed to nomads and in integrating animal and human health services. We also collaborated with field staff of the Chad Ministry of Livestock.

#### Developing a Menu of Intervention Options

A finding of the literature review was that interventions to reach nomadic populations with health services fell into 3 main categories: (1) offering joint delivery of animal or veterinary services, child immunization, and women’s health services [4, 5,17, 23], (2) building capacity of health centers or hospitals for mobility [24], and (3) recruiting and training local healthcare workers among nomads to vaccinate or organize vaccinations [7]. We considered the above intervention categories and included them in our intervention.

#### Identifying and Locating Nomadic Settlements

To reinforce our knowledge of the directions and timings of migratory movements by nomads, we held several meetings with nomadic community leaders, local government administrators, and veterinary service staff to identify corridors of transhumance that nomads used in our target areas (Table 2). To prepare for the 23–25 November polio vaccination campaign, for example, we held a meeting on 25 October 2012 in the district of Dourbali with local community leaders to collect information on their respective settlements, including global positioning system coordinates and mobile phone numbers. We also identified the 13 corresponding health areas or “zones of responsibility” in which the settlements were located and shared information with heath staff so they could include these settlements in their microplans. We collected similar information in all target areas during precampaign planning meetings. In the district of Massaguet, we identified a well-organized association of young and adult educated nomads—the Association des Jeunes Nomades Pour le Development Rural et la protection de L’Elevage au Tchad (AJNDRPET). We recruited and trained some of its members to conduct a census of both children less than 5 years of age and pregnant women who would be targeted with polio and tetanus vaccinations. Unfortunately, we were able to complete the census in only 5 of the 13 health zones because of logistical and time constraints.

#### Implementing Social Mobilization and Fostering Community Participation

Community leaders who participated early on in the project to help identify and locate their respective tribes also served as advocates and social mobilizers for vaccination of children against polio in their own communities. We recruited additional community leaders during weekly market days where many nomads came to trade. Key local figures, such as government appointed staff in the livestock sectors, were also brought on board at no cost and were effective in convincing nomads to accept vaccinations of their children, pregnant women, and animals. In the district of Massaguet, in particular, the AJNDRPET encouraged participation. Except for government staff, community members who participated in identifying and locating nomadic settlements or social mobilization activities received small monetary compensation.

#### Conducting Vaccination Activities

Vaccinations began in the postrainy season in November 2012, when nomadic populations in our intervention areas were moving westward, closer to water points around Lake Chad in the Lac region, as shown in Figure 1. Table 3 shows the calendar of vaccinations and target areas. We offered the bivalent oral polio vaccine to children 0–59 months of age. Particularly in the district of Massaguet, we offered the vaccine against tetanus to pregnant women, along with other child vaccinations recommended by the World Health Organization–EPI against tuberculosis, measles, meningococcal bacterium Neisseria meningitis group A, diphtheria, tetanus, whooping cough, hepatitis B, and Haemophilus influenzae type b. We also offered animal vaccinations against anthrax and blackleg. In the district of Kyabe, vitamin A was offered to children 6–59 months of age, in addition to the polio vaccine.

Except for the district of Massaguet, all vaccinations of nomads occurred during polio mass vaccination campaigns.

Vaccinations in Massaguet took place as part of a larger scheme to vaccinate animals during the seasonal crossing of nomads to Cameroon, where they customarily stay from January to June each year in search of grazing pastures [25, 26]. Veterinary services in Chad use this as an opportunity to vaccinate animals before the crossing.

Seizing the opportunity of this mass exodus, EPI, with support from partners, including the World Health Organization and United Nations Children’s Fund (UNICEF) Chad offices, also proposed to offer polio and other vaccines to children and women at the Waldoussou crossing point in the health zone of Djimtilo (see Figure 1). We used fixed posts to vaccinate during the cattle crossing event and also made arrangements between veterinarian and health staff in the surrounding health centers to place vaccination teams in axes before, at, and after the crossing point of Waldoussou on the Cameroon side of Lake Chad. Vaccination posts are shown in Figure 1.

Mobile teams of 2 persons traveled by motorcycle to nomadic settlements to vaccinate eligible children and pregnant women. Where possible, we recruited local adult nomads to be part of the vaccination teams and trained them to fill out tally sheets. In each team of vaccinators, ≥1 adult nomad could speak the language of the target tribes. We recruited veterinary staff at district levels to supervise vaccination teams, because they were well known among nomadic communities, familiar with the vaccination areas, and could serve as well-respected arbitrators in cases of vaccine refusals or resistance.

### Collecting and Analyzing Data

We collected vaccination coverage data during polio mass vaccination campaigns for all districts in the targeted regions except for Massaguet, where vaccinations were organized ad hoc to take advantage of the opportunity offered by the seasonal cattle crossing from Chad to Cameroon. Vaccination data consisted of (1) the number of nomadic children 0–59 months of age vaccinated, (2) the number of nomadic children vaccinated for the first time, (3) the number of nonnomadic children 0–59 months of age vaccinated, and (4) the number of nonnomadic children vaccinated for the first time.

We compared the number of nomadic and nonnomadic children vaccinated during mass campaigns before and after implementation of our interventions. The changes after the intervention were expressed as the percentage change from baselines. These relative changes were compared between intervention districts and nonintervention districts. We included all districts in the target regions and selected intervention districts on the basis of convenience; for example, our project coordinator had work experience in the selected districts, which helped him build easier linkages with local nomadic communities and administrative and technical staff in the areas. We present results from activities that took place between November 2012 and March 2013.

## RESULTS

### District of Dourbali, Chari-Baguirimi

As shown in Table 4, the number of vaccinated nomadic children increased in the intervention district of Dourbali from 2956 to 8164 (176% increase) but decreased in comparison districts, with the exception of Massenya. The number of vaccinated nonnomadic children also increased but to a much smaller extent (49 775 to 57 416; 15% increase). The number of nomadic children vaccinated for the first time increased by 118% as well in Dourbali but decreased by 42% among the nonnomadic children. In the comparison district, the total number vaccinated and the number vaccinated for the first time either decreased or increased by a small amount among both nomadic and nonnomadic children. The exception was the Mandelia district, where nomadic children showed an increase from 2 to 2740—the largest relative increase seen in any district.

### District of Kyabe, Moyen-Chari

The number of vaccinated nomadic children increased from 7319 to 15 868 (117% increase) in the intervention district of Kyabe but decreased by 4% in the comparison district of Danamadji and increased only by 7% in the comparison district of Sahr. The number of nomadic children vaccinated for the first time rose in the intervention district of Kyabe from 294 to 1738 (491% increase) and decreased in the comparison districts, by 36% in Danamadji and by 32% in Sahr (see Table 5).

### District of Massaguet, Hadjer-Lamis

Our main targets were the 5 health zones of N’Djamena Fara, Karal, Baltram, Guitte, and Mani and Djimtilo, chosen because of their location near the border with Cameroon. According to a local census conducted by our enumerators, about 5134 nomadic children 0–59 months of age were present in the 5 target health zones. This number represents 820 infants 0–11 months of age and 4314 children 12–59 months of age. Polio vaccination coverage during the crossing of cattle to Cameroon was estimated at only 37.7%. However, coverage was better among infants 0–11 months of age, with 67% (548 of 820) vaccinated, compared with only 32% of children 12–59 months of age. No baseline data were available on the target health zones.

## DISCUSSION

The improvement of polio vaccination coverage among nomads did not start with the implementation of our specific interventions but instead began when nomadic children up to 14 years of age were targeted for the first time in June 2012 during SIAs. As a result, our interventions may have had mainly an additive or enhancing effect to already improving polio vaccination coverage trends among nomads. Then again, the intervention districts were conveniently selected, and we therefore cannot generalize our results to other districts. Nevertheless, the strategy to target nomads during SIAs showed positive effects.

We observed increases in all intervention areas in the number of nomadic children vaccinated. The exception among the comparison districts was the district of Mandelia, which showed sharp increases without any interventions. We suspect this was due to accounting or recording errors at baseline, considering the strikingly low number of only 2 nomadic children vaccinated in October 2012, with none vaccinated for the first time during this month.

Polio vaccination coverage levels at the Waldoussou crossing were disappointingly low, despite the joint delivery of animal and other vaccines for both children and pregnant women. This was a unique vaccination opportunity, where large assemblies of nomads were present at a few points where they were easily accessible with certainty. We vaccinated fewer children, because not all families with eligible children who were identified by our enumerators crossed during the 10 days when we had teams in the fields. We withdrew vaccination teams after 10 days because of budget constraints. Some children may have left the area before the crossing. Another possibility is that the size of the target population used to estimate coverage may have been inflated by our enumerators. Although this study was not designed to address them directly, several factors are likely to have contributed to our ability to reach nomads with the polio vaccine. First, hiring a staff person dedicated to coordinate and manage the project was instrumental, because it provided oversight and accountability. The project coordinator also contributed local knowledge and field experience needed to effectively guide field operations and gain the trust of communities. Finally, the commitment of the national political and administrative leadership to eradicate polio in general created a favorable environment that led to the EPI’s full cooperation and its commitment of vaccine and staff resources.

Intersectoral collaboration was a useful and effective mechanism to attract nomadic families with in-demand services other than the polio vaccine, including vaccinations for their animals and immunizations against more common and feared childhood diseases such as measles and the tetanus vaccine for women. Finally, the vaccinators’ flexibility and capacity to adapt to circumstances and vaccinate when and where nomads were available or accessible may have also helped improve immunization rates. Because vaccinations took place during mass polio campaigns, little or no additional cost was associated with implementing targeted vaccinations to nomads, outside of the expenses usually incurred during regular SIAs.

A major limitation of this study is that the intervention was implemented in a convenient sample of areas that already had ties to the intervention team. Furthermore, the intervention was implemented in a small number of areas. Because accurate data are unavailable on the size of the nomadic population in Chad, it is impossible to say what proportions of the nomadic population were reached or affected in the target areas. In addition, it was impossible to assess the specific effects of the interventions on changes of vaccination coverage. This is particularly difficult to achieve with community interventions, because many external factors are in play. We did not implement projects at the same time in all areas. As a result, we have results for several months in some areas (eg, Chari-Baguirimi) and only for 1 month in others (eg, Moyen-Chari).

In conclusion, the response by Chad’s Ministry of Public Health, with support from its Global Polio Eradication Initiative partners to end the polio epidemic affecting nomads, consisted of targeting nomads as a distinct sociocultural and economic group during polio vaccination campaigns, using a mix of evidence-based interventions. Polio vaccination coverage of nomads increased particularly in areas where these targeted activities were implemented, although it also increased in 1 untargeted area where we suspect the remarkable improvement was due to a very low baseline. To our knowledge, factors that probably helped in developing and implementing the targeted vaccinations included (1) the appointment of a dedicated staff for coordination and oversight, (2) engagement by the national government and its partners, (3) the involvement of nomadic communities themselves, (4) the effective intersectoral collaboration between human and animal health services, and (5) the flexibility and capacity among vaccinators to adapt to circumstances and vaccinate when and where nomads were available or accessible, according to their seasonal movements and timing of life events.

## Figures and Tables

**Table 1.** Results of Literature Review: Short List of Interventions Targeting Nomadic Populations

References	Targets	Country/Setting	Strategy/Intervention	Implementation Strategy (Key Components)	Outcomes	Challenges for Implementation and Limitations of Strategy
Schelling [4], Schelling et al [16], Schelling, et al [17] (polio included)	Nomadic pastoralist children and women (Fulani, Arabs, Dazagada) livestock	Chad: Chari-Baguirimi and Kanem	Offer joint mobile veterinary and public health services; multiple services	Engage heads of camps; develop information materials on vaccination (pictograms) in local language to enhance awareness; harmonize timing of activities between veterinarians and public health services; include women’s health (eg, TT and prenatal care, etc.); organization of 3 round campaigns	4700 children <5 y old fully vaccinated; 7700 women receive ≥2 TT doses; low cost of public health sector (15% of operating costs); 131 (95% CI, 115–148) persons vaccinated in 176 joint vaccination days vs 100 persons vaccinated (95% CI, 94–106) in 377 human-only vaccination days	Availability of vaccines, limited government infrastructure; difficulty to travel/locate targets in wet season; denominator not known; failure to adhere to planned schedule of immunization rounds; lack of advance publicity and preparations of the populations; inadequate motivation of personnel; large area to cover
Belmaker et al [18] (polio included)	Seminomadic Bedouin Arabs	Israel, Negev desert	Use mobile immunization teams and increase access to MCH with more staff and more MCH clinics	Recruit local Bedouin women as community liaisons; mobile team with nurse and Bedouin driver; centralized database to track unimmunized children	Measles coverage increased from 53% to 90% among children <2 y old, 1992–2003; no cases of polio despite an epidemic in Egypt in 2002	Cost of mobile clinics^a^ and integration of teams into routine care of MCH
Brieger et al [8] (Guinea worm, surveillance)	Fulani	Nigeria, Ifeloju LGA, Oyo State	Active search and listing of settlements and enrollment of local leaders; recruit and train local community health workers for surveillance	Use of LGA baseline list complemented through collaboration with UNICEF project to locate borehole wells used by Fulani herders	More cases of Guinea worms reported, mostly from formerly unidentified settlements (same findings in Bornu project, 2005)	Cultural barriers (not good relations between LGA staff and Fulani for integration into the routine surveillance system)
Sheik-Mohamed and Velema [7] (primary healthcare)	Sub-Saharan nomads	Cross-country/international review	Recruitment and training of nomadic healthcare workers	Nomadic healthcare workers live and move with nomads; register eligible children and schedule vaccination sessions in nearest village with EPI teams; allowed to get drugs at any health center (regular POC along migration routes).	None reported; strategy not actually implemented but improved primary healthcare suggested	Political will to provide sustained and effective support; government interference
Zinsstag et al [5] (primary healthcare)	Nomadic population (general)	Sub-Saharan Africa	Use of mobile clinics and transsectoral approaches between animal and public health		None reported; strategy not actually implemented but improved vaccination coverage is suggested	Mistrust, low perception of health priorities by nomads and preference for traditional medicines; community participation
Sani [19] (EPI program)	Pastoralist	Agadez region, Niger	Mobility capable fixed structures with seasonal circuits in defined operational areas	Carry out integrated fixed and mobile activities in defined operational areas	None reported; strategy not actually implemented but improved overall; EPI program is suggested	None reported
Shears [20] (surveillance)	Nomadic population	West Africa	Integrate human disease surveillance to animal disease surveillance framework of international organizations^b^			
Borno state team [21]	Fulanis and Arabs	Borno, Nigeria	Temporary fixed vaccinating sites and mobile teams	Location/listing of camps, community participation, multiple service provisions	73% OPV coverage	Duration of activities, vaccine wastage

Abbreviations: CI, confidence interval; EPI, Expanded Program on Immunization; LGA, Local government area; MCH, Maternal Child Health; OPV, oral polio vaccine; POC, Point of contact; TT, tetanus toxoid; UNICEF, United Nations Children’s Fund.

a In Mali, the cost per fully immunized nomadic child with mobile vaccination teams was found to be 11 times higher than for settled individuals (Imperato [9]). The cost per fully immunized nomadic child is 2.3 times higher than for static facility services in different African countries (Brenzel and Claquin [22])

b International organizations include the Pan African Programme for Control of Epizootics, the Emergency Prevention System for Trans-boundary Animal and Plant Pests and Diseases, and the Regional Animal Disease Surveillance and Control Network.

**Table 2.** Corridors of Transhumance of Nomads in the Target Regions

Region	Corridors of Transhumance
Chari-Baguirimi	
Bokoro	Axis: Bokoro toward Karme, Ngoura, and Moito
Dourbali-Massenya	Axis 1: Balday-Djinhere-Abgarga-Balao-Karnak-Massenya Axis 2: Billi-Badjoda-est Massenya-Moudou-Boudamassa-Bousso Axis 3: Dourbali-Bodo-Katchi-Tinguil toward Barh Rigueygue or Chari
Hadjer-Lamis	
Massaguet	Axis 1: Massaguet-Djerma-N’Djamena Fara-Douguia-Hadide or Nibec or Amdarbaya then crossing to Cameroon. Axis 2: Birbarka-Matchi-Balbout-Drug-Baltram-Meterine, then to island areas (Kasanlare-Nara-Katikime, Kodjirom . . .) Axis 3: Eastward movement from Massaguet toward north of Dourbali or N’Djamena
Massakory	Axis 1: East Massakory-Amchoka-Karme Axis 2: Kamerom-Tourba-Gredaya-Karal Axis 3: Massakory-Massaguet
Moyen-Chari	
Districts of Bousso-DRS Tandjile-Mandoul	Axis 1: Bousso-Kilala-Goundi-Garanga-Mouroumgaye-Koko-Peni-valley of Mandoul-Bekourou (international border crossing point to Markounda, (CAR) Axis 2: Bousso-Kouno-Kira-Bedaya-valley of Mandoul-Bekourou (international border crossing point to Markounda (CAR)
Melfi-DRS	Sila-Melfi-Daguila-Korbol-Mabrouka-Sido (international crossing point to CAR)

Abbreviations: CAR, Central African Republic; DRS, Direction Regional de la Sante.

**Table 3.** Calendar of Polio Vaccination Campaigns After Interventions and Targeted Districts

Dates of Vaccination Campaign	Region	Districts
23–25 November 2013	Chari-Baguirimi	Intervention: Dourbali; comparison: Massenya, Bousso, and Mandelia
8–17 January 2013	Hadjer-Lamis	Intervention: Massaguet^a^
15–17 March 2013	Moyen-Chari	Intervention: Kyabe; comparison: Danamadji and Sahr

a No preintervention and comparison data were available.

**Table 4.** Vaccination Performance in Intervention and Comparison Districts, Region of Chari-Baguirimi

	Children Vaccinated, No.		
Target Group	October 2012 (Baseline)	March 2013 (End Period)	Change, %
District of Dourbali (intervention)			
Nonnomadic children	49 775	57 416	+ 15
Nomadic children	2956	8164	+ 176
Nonnomadic children (1st vaccination)	566	326	−42
Nomadic children (1st vaccination)	60	131	+ 118
District of Bousso (comparison)			
Nonnomadic children	56 682	58 832	+4
Nomadic children	3215	924	−71
Nonnomadic children (1st vaccination)	4569	1452	−7
Nomadic children (1st vaccination)	394	93	−76
District of Mandelia (comparison)			
Nonnomadic children	35 949	37 937	+5
Nomadic children	2	2740	^a^
Nonnomadic children (1st vaccination)	995	591	−40
Nomadic children (1st vaccination)	0	76	^a^
District of Massenya (comparison)			
Nonnomadic children	28 892	29186	+1
Nomadic children	6833	4716	−31
Nonnomadic children (1st vaccination)	537	447	−17
Nomadic children (1st vaccination)	191	210	+10

a The percentage change is too big (many thousand–fold) owing to a very low or near-zero baseline.

**Table 5.** Vaccination Performance in Intervention and Comparison Districts, Region of Moyen-Chari

	Children, No.	
Target groups	February 2012 (Baseline)	March 2012 (End Period)	Change, %
District of Kyabe (intervention)			
Nonnomadic children	50 222	50 636	+ 1
Nomadic children	7319	15 868	+ 117
Nonnomadic children (1st vaccination)	1008	1235	+23
Nomadic children (1st vaccination)	294	1738	+491
District of Danamadji (comparison)			
Nonnomadic children	67 482	68132	+1
Nomadic children	5847	5599	−4
Nonnomadic children (1st vaccination)	1087	704	−35
Nomadic children (1st vaccination)	171	109	−36
District of Sahr (comparison)			
Nonnomadic children	79 610	79 529	−0.10
Nomadic children	4114	4402	+7
Nonnomadic children (1st vaccination)	1127	713	−36
Nomadic children (1st vaccination)	185	126	−32
